# Additive protection of transcranial direct current stimulation and mitochondrial transplantation on ischemic stroke via inducing A2 astrocytes

**DOI:** 10.3389/fnmol.2026.1855078

**Published:** 2026-09-09

**Authors:** Yaomin Guo, En-ming Kang, Min Li, Taozhi Wang, Zhongqing Sun, Yazhou Wang, Wen Jiang, Jiang Li

**Affiliations:** 1 Department of Neurology, Xijing Hospital, Fourth Military Medical University, Xi’an, Shaanxi, China; 2 Department of Neurobiology, School of Basic Medicine, Fourth Military Medical University, Xi’an, Shaanxi, China; 3 The Shaanxi Key Laboratory of Brain Function Analysis and Modulation, Xi’an, Shaanxi, China; 4 Department of Neurobiology, Medical College of Yan’an University, Yan’an, China; 5 Department of Anesthesiology and Perioperative Medicine, Xijing Hospital, The Fourth Military Medical University, Xi’an, Shaanxi, China; 6 Department of Neurosurgery, Tangdu Hospital, Fourth Military Medical University, Xi’an, Shaanxi, China

**Keywords:** astrocytes, endocytosis, ischemic stroke, mitochondria, tDCS, transplantation

## Abstract

**Introduction:**

Therapeutic options for the acute phase of ischemic stroke remain limited. Transcranial direct current stimulation (tDCS) and mitochondrial transplantation have emerged as promising neuroprotective approaches, but their individual efficacy is variable. We hypothesized that combining these two therapies would produce additive benefits for post-stroke recovery.

**Methods:**

Focal cortical ischemia was induced in mice using photothrombotic technique. Mice were randomly assigned to receive sham treatment, tDCS, mitochondrial transplantation, or combined treatment. Grid-walking, cylinder tests and 2,3,5-triphenyltetrazolium chloride staining were used to assess motor recovery and infarct volume, respectively. Immunofluorescence staining, and western blotting were performed to determine mitochondrial internalization and polarization of astrocytes *in vivo* and *in vitro*.

**Results:**

Combining tDCS with mitochondrial transplantation resulted in a significantly greater reduction in infarct volume and improvement in locomotor function compared to either treatment alone. Interestingly, tDCS specifically enhanced the uptake of exogenous mitochondria by astrocytes. This was associated with a significant increase in beneficial A2 astrocytes and decrease in detrimental A1 astrocytes. Mechanistically, the combined treatment led to a marked upregulation of CD38 in astrocytes, suggesting their involvement in the tDCS-facilitated mitochondrial endocytosis. Suppression of CD38 expression by short interfering RNA attenuated astrocyte mitochondrial endocytosis and A2 phenotype induced by tDCS.

**Conclusion:**

Our findings demonstrate that combining tDCS with mitochondrial transplantation conferred superior neuroprotection against ischemic brain damage than either treatment alone, and may represent a promising strategy for ischemic stroke treatment.

## 1 Introduction

Ischemic stroke (IS) is a major cause of death and disability worldwide and continues to pose a significant global health burden. Decades of research have been devoted to improving the understanding of its pathophysiology and developing interventions and rehabilitation (Feigin et al., 2020; GBD 2021 Diseases and Injuries Collaborators, 2024). Currently, most effective treatments for ischemic stroke focus on acute phase, involving endovascular intervention, intravenous thrombolysis and antiplatelet therapy (Sharma and Lee, 2025). However, the short time-window and strict inclusion criteria make only few patients can receive effective interventions timely after the onset of ischemic events (Diaz et al., 2026). Therefore, developing novel therapeutic strategies which could complement or provide alternatives to acute stroke interventions is crucial to promote restorative processes.

Transcranial direct current stimulation (tDCS) is a noninvasive brain stimulation technique, which can reversibly modulate the neuronal activity in particular brain regions by current flows between the electrodes (Bikson et al., 2016). Previous studies have demonstrated that, when applied to IS, tDCS exerted multiple beneficial effects such as regulating cortical excitability (Cirillo et al., 2017), anti-apoptosis (Huang et al., 2025), diminishing glial activation (Zhang et al., 2020), and enhancing dendritic plasticity and axonal regrowth (Longo et al., 2022). However, the outcome of tDCS treatment showed notable variation in different researches, leaving its clinical application obscure (Markowska and Tarnacka, 2024).

One hallmark of ischemia-induced pathology is the mitochondrial dysfunction in multiple cells (Yang et al., 2018). Recently, researchers revealed a phenomenon called intercellular mitochondrial transfer which confers protective effects by supporting cell survival, facilitating ATP generation, rebalancing metabolic program, and even regulating cell fate (Nakamura et al., 2020; Tait and Green, 2012). This observation suggests that exogenous mitochondria transplantation may be as a potential therapy to promote brain repair. An increasing body of study suggests mitochondrial transplantation can significantly improve motor function and reduce infarct size by promoting the neuronal viability, refreshing damaged mitochondria, decreasing cellular oxidative stress, and suppressing apoptosis in mice stroke models (McCully et al., 2016; Huang et al., 2016). Unfortunately, the internalization of exogenous mitochondria is relatively weak in adult brain, thus limiting the beneficial effects of mitochondrial transplantation (Zhou et al., 2025).

Considering that mitochondria-skeleton interaction is sensitive to electrical field (Reynaud et al., 1989; Goswami et al., 2018; Qi et al., 2019), we hypothesized that combination of tDCS with mitochondrial transplantation would produce additive effects. The present study is aimed to investigate this possibility. Our data showed that combining mitochondrial transplantation with tDCS conferred superior neuroprotection against ischemic brain damage than either treatment alone.

## 2 Materials and methods

### 2.1 Animals

Adult males C57BL/6 mice, weighted 20–25 g, aged 6–8 weeks, were obtained from the animal facility of the Fourth Military Medical University. All mice were acclimatized for at least 1 week under humidity- and temperature-controlled condition with a 12 h dark/light cycle. All experimental procedures were performed in accordance with ARRIVE guidelines and approved by the Committee of the Animal Care and Use of the Fourth Military Medical University (No. KY20254136-1). In accordance with animal welfare guidelines, measures to alleviate animal suffering and reduce the number of animals used were adopted, and the sample size was chosen according to previous literatures to minimize animal numbers while maintaining statistical validity (Longo et al., 2022; Salman et al., 2024).

### 2.2 Photothrombotic ischemia of mice cortex

Focal cortical ischemia was induced in anesthetized mice using the photothrombotic technique. Rose Bengal (Solarbio, Cat#G8540) was dissolved in sterile 0.9% saline with a concentration of 10 mg/mL and administered via tail vein injection. Subsequently, a cranial window was carefully created on the 0.5–3.0 mm lateral to the midline of skull, ensuring the brain tissue remained unharmed. Within 15 min after the injection, the exposed area was illuminated for 8 min using a cold light source (Zeiss KL1500 LCD, 45–50 mW) to induce the photothrombotic reaction (Fan et al., 2022).

### 2.3 Transcranial direct current stimulation

A programmable electrical stimulation device (Jinan Yiyan Science & Technology Development Co., Ltd., China) was used to deliver tDCS. Given the critical time window for mitochondrial uptake and acute post-stroke risks (intracranial pressure fluctuations, cerebral edema, and effects of anesthetic agents), the tDCS was performed following a 12-h post-stroke stabilization period. During the treatment, the cathodal of tDCS was positioned over the ipsilesional motor cortex, while the anode was applied over the contralesional side. The protocol consisted of a daily 20-min session delivered at a current intensity of 250 μA in 3 consecutive days (Longo et al., 2022). The sham stimulation protocol mimics the same manipulations as “real” stimulation procedure, without delivering current. No adverse events were observed during the treatment.

### 2.4 Neurological function tests

Functional outcome after cortical ischemia was evaluated by grid-wallking test and cylinder test (Syeara et al., 2023). The foot fault test was conducted on an elevated 50 cm height horizontal ladder with 2.5 cm between rungs. A step was defined as a foot-fault when the limb slipped or missed a rung, thereby failing to provide support. The foot-fault percentage was calculated as the following formula: (number of foot faults/total number of steps) × 100%, based on a 5-min recording period.

In the cylinder test, mice were placed in a transparent glass cylinder for 10 min, recorded from a bottom-up view to observe the mouse’s activity. The number of rearings was counted and categorized as each forelimb rearings involving only left (contralateral) forelimb, only right (ipsilateral) forelimb, and bilateral forelimbs. The percentage of rearings events of right forelimb was calculated as the sum of right rearings events divided by the total rearings events (right + 0.5 × bilateral)/(right + left + bilateral), termed as affected forelimb use index.

### 2.5 CD38 siRNA experiments

Two days before modeling, mice received a local intracortical injection of mouse Cd38 siRNA or control siRNA, a pool of three target-specific 19-25 nucleotide siRNAs with sequences: 5’-GUGUACUACCAACAUUCAA-3’, 5’-GUGUGUCUUUAGUAGGUAU-3’, and 5’-CCAGUUUGUGAUUGUUGA-3’, accroding to a previous study (Hayakawa et al., 2016).

### 2.6 Mitochondrial isolation, labeling and transplantation

Mitochondrial isolation was performed using a commercial extraction kit (Solarbio, Cat#SM0020) following the provided protocol. Briefly, 100 mg of freshly liver tissue from anesthetized wild-type mice was immediately rinsed with ice-cold PBS and subjected to mechanical homogenization. Tissue disruption was performed in a pre-cooled glass homogenizer containing 1.0 mL of ice-cold lysis buffer with approximately 30 strokes. Then, a series of differential centrifugation steps were carried out to achieve mitochondria purification. Two consecutive low-speed centrifugation steps at 1,000×*g* (5 min, 4°C) were employed to remove unbroken cells, cellular nuclei and debris within the cellular homogenatere, leaving the mitochondria in the supernatant. Subsequently, a high-speed centrifugation (12,000×*g*, 10 min, 4°C) was used to pellet mitochondria from supernatants. To further enhance purity, 0.5 mL of pre-cooled wash buffer were added to resuspend the mitochondria pellet and followed by a two steps centrifugation (1,000×*g*, 5 min to remove aggregates, then 12,000×*g*, 10 min, both 4°C). In order to track the mitochondrial translocation and spatial distribution, we applied MitoTracker Red CMXRos probe (Solarbio, Cat# C1035) to incubate with isolated mitochondrial fraction for 20 min. After incubation, the mitochondrial suspension was washed once again to remove any unbound dye. Throughout the procedure, strict aseptic techniques were maintained to avoid contamination.

For mitochondrial transplantation, the amounts of mitochondria transplanted were determined as described (Li et al., 2025). Briefly, freshly isolated mitochondria were diluted to 80 mg/mL and a suspension 0.5 μL (40 μg) freshly isolated mitochondria were labeled with MitoTracker and directly injected into the brain cortex. This was completed within 1 h after photo-thrombosis injury. For *in vitro* experiments, labeled mitochondria were added to the culture medium of primary astrocytes after 6-h oxygen glucose deprivation (OGD) and co-incubation for 24 h. Mitochondrial retention was assessed after 24 h using immunofluorescence techniques.

### 2.7 2 ,3,5-triphenyltetrazolium chloride (TTC) staining

Three days after mitochondrial transplantation, as well as the 24 h after the final tDCS treatment, mice were sacrificed. The isolated brains were cut using a pre-cold brain slicer (RWD, China) into 100 μm thick coronal slices. From each brain, five representative sections were obtained. The sections were incubated in a 2% TTC solution (Solarbio, Cat#G3005) for 20 min at room temperature in the dark and imaged using a camera. The ischemic size was determined and quantified with ImageJ software (https://imagej.net/ij/), which were expressed as the ratio of the ischemic areas to the total brain section areas. Considering the effect of edema, the corrected ratio of infarct volume was calculated using the following formula: (contralateral hemisphere volume − ipsilateral hemisphere TTC-unstained volume)/total brain volume × 100%.

### 2.8 Nissl staining

For Nissl staining assay, the sections were dehydrated, added dropwise with Nissl reagent (Beyotime Biotechnology, Shanghai, China) and stained for 30 min. Afterwards, the sections were washed with tap water, dehydrated with alcohol, and treated with xylene, followed by observation under a light microscope.

### 2.9 TUNEL staining

The apoptosis of neurons in mice brain tissues was detected using TUNEL staining according to the manufacturer’s instructions of commercially available kit (HY-K1078; MedChemExpress, USA). The numbers of TUNEL-positive and total neurons were counted, and apoptosis was evaluated by the ratio of positive cells to total cells.

### 2.10 Primary astrocytes culture, oxygen-glucose deprivation, and mitochondria/DCS treatment

Primary astrocytes were isolated from the cerebral cortical of 3-day-old neonatal C57BL/6 mice. The cortical tissue was enzymatically dissociated using 0.25% trypsin in Dulbecco’s Modified Eagle Medium (DMEM, Gibco, COT#11965092) for 10 min at 37°C, followed by centrifugation at 800 × g for 10 min. The cell pellet was resuspended in complete culture medium, containing DMEM, 10% fetal bovine serum (Gibco, Cat#10099141C), 4 mM L-glutamine (Gibco, Cat#25030081), and 1% penicillin/streptomycin (Gibco, Cat#15070063) and then plated onto a 75 cm^2^ culture flasks. After the cells reached 90–100% confluency, the flasks were shaken at 260 rpm for 16–18 h to removed non-astrocytic cells. The adherent astrocytes were trypsinized and replated for further experiment.

In the OGD group, primary astrocytes were washed 3 times with PBS and changed the culture medium to glucose-free DMEM (Gibco, Cat#11966025). The plates were placed in a 37°C chamber with anaerobic gas conditions (5% CO_2_ and 95% N_2_) for 6 h. In the OGD + Mito and OGD+direct current stimulation (DCS) + Mito group, isolated exogenous fresh mitochondria were added along with the complete medium immediately after reoxygenation. In the OGD + DCS and OGD+DCS+Mito group, cells were were subjected to DCS using a vitro stimulation chamber, applying a constant voltage of 2.2 V (100 mV/mm) for 10 min (Mobini et al., 2016; Srirussamee et al., 2021).

### 2.11 Mitochondrial membrane potential measurement

Mitochondrial membrane potential was measured following the manufacturer’s protocol provided by the JC-1 MMP assay Kit (Beyotime, Cat#C2006). After treating the cells for 24 h with mitochondria/DCS, 1 mL JC-1 working solution (10 μg/mL) was added to each group and incubated at 37°C for 20 min. After washing with buffer solution and adding fresh culture medium, the live cells were imaged using a confocal laser scanning microscope. The fluorescence detection channels were defined as follows: excitation at 490 nm and emission at 530 nm for the JC-1 monomer; excitation at 525 nm and emission at 590 nm for JC-1 polymer. The mean fluorescence intensity (MFI) of each image was measured using ImageJ software.

### 2.12 Immunofluorescence

Mice were sacrificed and immediately perfused intracardially with pre-chilled 4% paraformaldehyde (PFA). The brain tissue was dissected after perfusion and post-fixed by 4% PFA for about 2 h at 4°C. Then, the brain was cryoprotected in 30% sucrose solution until complete sank. Free-floating 20 μm coronal sections were cut on a cryostat (RWD, China). The frozen brain sections were washed with PBS, blocked and permeabilized with 3% BSA and 0.3% Triton X-100 for 1 h. Primary antibodies were incubated overnight (16–18 h) at room temperature. Primary antibodies included aldehyde dehydrogenase 1 family member L1 (ALDH1L1, 1:200, Proteintech, 17390-1-AP) neuron-specific nuclear protein (NeuN, 1:200, Proteintech, 66836-1-lg), ionized calcium binding adaptor 1 (Iba-1, 1:200, abcam, ab5076), Complement 3 (C3, 1:200, Proteintech, 21337-1-AP), S100 calcium binding protein A10 (S100a10, 1:200, Proteintech, 11250-1-AP), glial fibrillary acidic protein (GFAP, 1:200, Invitrogen, 13-0300), cluster of differentiation 38 (CD38, 1:200, Proteintech, 25284-1-AP), and sigma-1 receptor (Sig-1R, 1:200, Proteintech, 15168-1-AP). Sections then were washed with PBS and incubated with secondary antibodies (1:800, Abcam) for 2 h at room temperature, followed by DAPI staining.

Images were acquired using a confocal microscope (FV3000, Olympus, Tokyo, Japan). Cell counting was performed by an investigator blinded to the experimental design. The ischemic region was defined as the infarct area with marked morphological changes, identified by comparison with the contralateral cortex. For quantification, cells were counted across the entire ischemic region in eight serial sections at regular intervals; specifically, cell numbers in the ischemic region were determined from 6 randomly selected coronal sections per mouse, with at least three mice included in each experimental group. The percentages of specific marker-positive cells, presented as histograms in the figures, were calculated using the formula: (number of specific marker-positive cells/total cell count) × 100%.

### 2.13 Western-blotting

Total proteins were extracted from the ipsilateral per-infarct cortices and its concentration were quantified using a bicinchoninic acid protein (BCA) assay kit. The protein samples were denatured by boiling at 95°C for 10 min. Equal amounts of protein (20 μg) were separated by SDS-PAGE on 10% gradient gels and transferred to 0.22 μm PVDF membrane. The membranes then were blocked with 5% nonfat milk solution for 2 h at room temperature and incubated overnight at 4°C with the primary CD38 antibody (1:1000, Proteintech, 25284-1-AP) and Sig-1R antibody (1:1000, Proteintech, 15168-1-AP) followed by incubation with corresponding horseradish peroxidase (HRP)-conjugated secondary antibodies at room temperature for 2 h. The protein bands were finally observed under a gel imaging system. The uncropped images of all Western blots are provided in Supplementary Datasheet 1.

### 2.14 QRT-PCR

Total RNA was isolated from ischemic cortex using TRIzol reagent (Invitrogen). RNAs were reverse transcribed with a PrimeScript RT reagent kit (TaKaRa, Tokyo, Japan). qRT- PCRs were performed in triplicate to detect the expression of *C3* and *S100a10* with SYBR Premix Ex Taq II (Takara) using a CFX96 Real-Time PCR Detection System (Bio-Rad, Hercules, CA, USA). The relative quantification of each genewas normalized to *β-actin* and calculated by the 2^–Δ^ ^Δ^ *^Ct^* method (Fei et al., 2022). Primers sequences were as follows:

*C3*-Forward: AACAAGCTCTGCCGTGATG,

*C3*-Reverse: GCCTGACTTGATGGTCTGCTCT,

CD38-Forward: TCTCTAGGAAAGCCCAGATCG,

CD38-Reverse: GTCCACACCAGGAGTGAGC,

*S100a10*-Forward: TGAGAGTGCTCATGGAACGG,

S100a10-Reverse: AGAAAGCTCTGGAAGCCCAC,

*β-actin*-Forward: GAGACCTTCAACACCCCAGC,

*β-actin*-Reverse: ATGTCACGCACGATTTCCC.

### 2.15 Statistical analysis

Statistical analyses were performed using GraphPad Prism (version 10.4.1; GraphPad, San Diego, CASoftware, USA). Quantitative data were normally distributed and presented as mean ± standard error of the mean (SEM). The statistical differences between groups were analyzed by using the Student’s *t*-test if comparing two groups. For multi-groups analysis, two-way ANOVA was used for behavior test and one-way ANOVA was used in other assays. When the ANOVA result was significant, Tukey’s honestly significant difference (HSD) test was performed to determine the differences between specific groups. *P* < 0.05 was taken as statistically significant.

## 3 Results

### 3.1 tDCS combined mitochondrial transplantation exhibits additive protection on ischemic stroke

As previously described, we conducted unilateral photothrombotic ischemia which produces consistent injury area (Fan et al., 2022). For tDCS, we used a widely adopted protocol (250 μA, 20-min, 3 consecutive days) (Longo et al., 2022). For mitochondrial transplantation, we adopted a protocol which has been successfully applied in traumatic brain injury (Zhao et al., 2021). The viability and respiratory complex activity of were confirmed by Mitotracker staining and enzymic activity (Supplementary Figure 1). Mice were randomly assigned to the IS, IS + tDCS, IS + exogenous mitochondria (Mito), or IS + tDCS + Mito group and subjected to sham treatment, bi-hemispheric tDCS, mitochondrial transplantation or bi-hemispheric tDCS combined mitochondrial transplantation, respectively.

**FIGURE 1:**
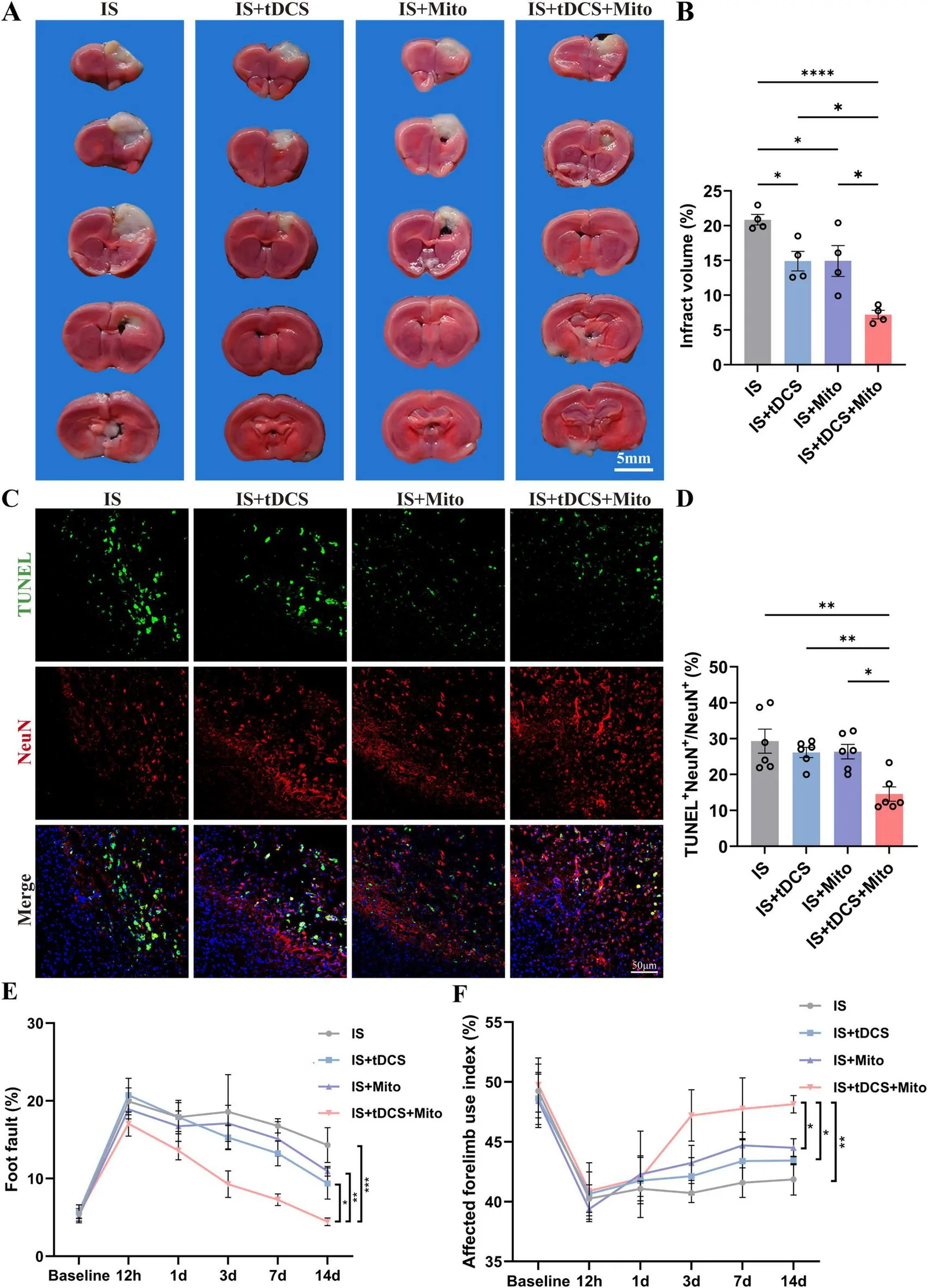
Effects of ischemic (sham treatment), tDCS, Mito, and combined tDCS+Mito treatments on ischemic lesion and functional recovery. **(A, B)** Representative results of TTC staining and quantitative analysis of infarct volume ratio at 3 pdi (*n* = 4 mice per group, F(3, 12) = 15.87, *P* = 0.0002). **(C, D)** Representative immunofluorescence images of TUNEL/ NeuN staining and quantification of apoptotic neurons (TUNEL^+^NeuN^+^ positive cells) at 3 dpi (*n* = 6 mice per group, F(3, 20) = 7.99, *P* = 0.0011). **(E)** Assessment of gait coordination using the grid-walking test (*n* = 6 mice per group, F_time_ (5, 115) = 30.64, *P* < 0.0001, F_treatment_ (3, 20) = 12.24, *P* < 0.0001). **(F)** Evaluation of forelimb use asymmetry via the cylinder test (*n* = 6 mice per group, F_time_ (5,115) = 12.16, *P* < 0.0001, F_treatment_ (3,20) = 3.28, *P* = 0.0422). All data are presented as mean ± standard error of the mean (mean **± SEM)**. Statistical significance was indicated as follows: *P < 0.05, **P < 0.01, ***P < 0.001, ****P < 0.0001. IS, ischemic stroke. tDCS, transcranial direct current stimulation. Mito, mitochondrial transplantation.

Morphological analysis was performed at 3 days post injury (dpi) when largest infarct area was found in IS group. Relative quantification revealed significantly smaller infarct volume in IS + tDCS and IS + Mito group, as compared with IS group (Figures 1A, B), suggesting a protective effect of tDCS treatment and Mito transplantation alone. No significant difference was observed between IS + Mito and IS + tDCS group. IS + tDCS + Mito group showed the smallest infarct volume among all groups (Figures 1A, B). TUNEL staining revealed predominant TUNEL+ cells along the margin of lesion area in IS group (Supplementary Figure 2), suggesting a hint of secondary injury. We thus focused on the ischemic penumbra for detailed analysis. Combination of TUNEL staining and immunohistochemistry showed that neither tDCS or Mito treatment alone reduced the TUNEL/NeuN double-positive cells, as compared with that in IS group (Figures 1C, D). Only IS + tDCS + Mito group showed significantly smaller number of TUNEL/NeuN double-positive cells as compared with IS group (Figures 1C, D). In similar, Nissel staining also showed the IS + tDCS + Mito group retained the highest number of viable neurons in the ischemic penumbra (Supplementary Figure 3).

Grid-walking test and cylinder test were performed at 12 h, 1, 3, 7 and 14 dpi to evaluate motor deficits. In grid-walk test, we observed partial spontaneous motor recovery in IS group as the proportion of foot faults decreased progressively over time. IS + tDCS and IS + Mito group showed a faster recovery compared to the IS mice, with no significant difference between these two groups. Remarkably, IS + tDCS + Mito achieved the best motor functional improvement among all groups with the consistently lower percentage of foot fault at every time points we observed (Figure 1E).

Cylinder test showed obvious deficits of forelimb motor function, with negligible recovery observed throughout the 14-day experimental duration (Figure 1F). The use index of affected forelimb in IS + tDCS and IS + Mito is significantly higher than that of ischemia group. Notably, the use index in the combined therapy groups is the highest among all groups (Figure 1F).

These results indicated that individual tDCS or Mito treatment is beneficial for the focal ischemic stroke. Combining tDCS with Mito application can achieve an additive protection leading to the superior function outcomes following ischemic stroke.

### 3.2 tDCS facilitates the internalization of exogenous mitochondria by astrocytes

**FIGURE 2:**
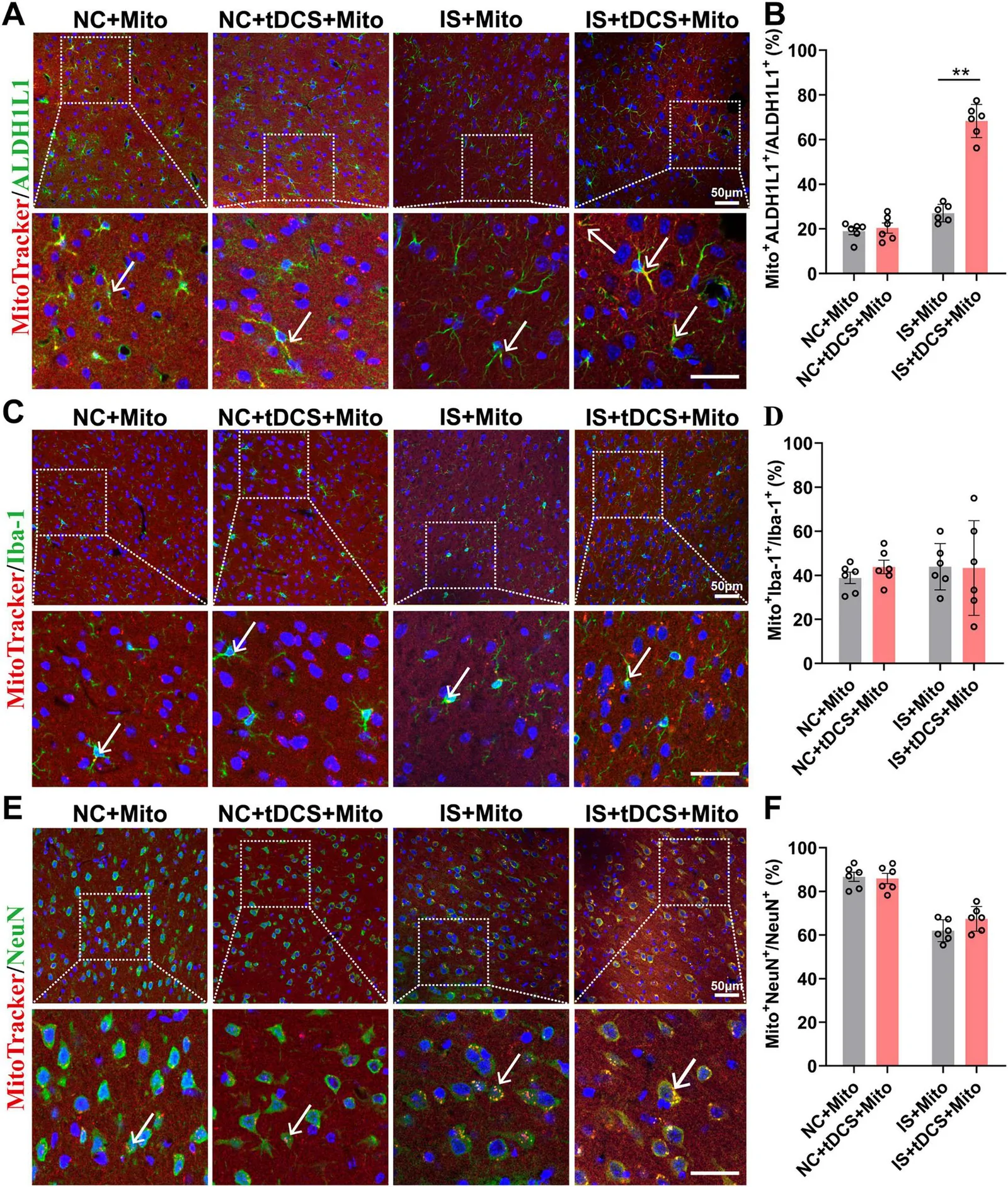
Enhancement of astrocytic internalization of exogenous mitochondria by tDCS. **(A, B)** Representative immunofluorescence images and quantitative analysis of MitoTracker^+^/ALDH1L1^+^ double positive cells in NC+Mito, NC+tDCS+Mito, IS+Mito, IS+tDCS+Mito groups (*n* = 6 mice per group). **(C, D)** Representative immunofluorescence images and quantitative analysis of MitoTracker^+^/Iba-1^+^ double positive cells in NC+Mito, NC+tDCS+Mito, IS+Mito, IS+tDCS+Mito groups (*n* = 6 mice per group). **(E, F)** Representative immunofluorescence images and quantitative analysis of MitoTracker^+^/NeuN^+^ double positive cells in NC+Mito, NC+tDCS+Mito, IS+Mito, IS+tDCS+Mito groups (*n* = 6 mice per group). Scale bars = 50 μm. Data are presented as mean ± standard error of the mean (mean ± SEM). Statistical comparisons were performed separately within each condition (NC or IS) using unpaired two-tailed Student’s *t* test. Statistical significance was indicated as follows: **P < 0.01; NC, non-injured cortex. IS, ischemic stroke. tDCS, transcranial direct current stimulation. Mito, mitochondrial transplantation.

We next explored how tDCS + Mito produced additive effects. According to our hypothesis, we assessed the cellular internalization of exogenous mitochondria in different neural cell types *in vivo* at the presence or absence of tDCS. Immunostaining demonstrated that pre-labeled exogenous mitochondria were clearly detected in neural cells around the infarct area in all groups received mitochondrial transplantation. In non-injured cortex (NC) treated with mitochondrial transplantation (NC + Mito), and mitochondrial transplantation combining tDCS (NC + tDCS + Mito), there was no difference in the efficiency of mitochondrial uptake in ALDH1L1-postive astrocytes, Iba-1-positive microglia and NeuN-positive neurons (Figure 2). Strikingly, in ischemic cortex, the proportion of ALDH1L1-postive cells containing internalized mitochondria was approximately 2.5-fold higher in the IS + tDCS + Mito group (62%) than in the IS + Mito group (25%, Figure 2A,B). Besides, approximately 40% of Iba-1-positive cells (Figure 2C,D) and 60% of NeuN-positive cells (Figure 2E,F) contained MitoTracker-labeled mitochondria. The efficiency of mitochondrial uptake in NeuN-positive neurons or Iba-1-positive microglia did not differ significantly between the IS + Mito group and IS + tDCS + Mito group (Figure 2D,F). These data demonstrate that tDCS can specifically enhance the uptake of exogenous mitochondria by astrocytes in the ischemic brain.

### 3.3 tDCS combined with exogenous mitochondria bolsters A2-like while suppressing A1-like astrocytes

**FIGURE 3:**
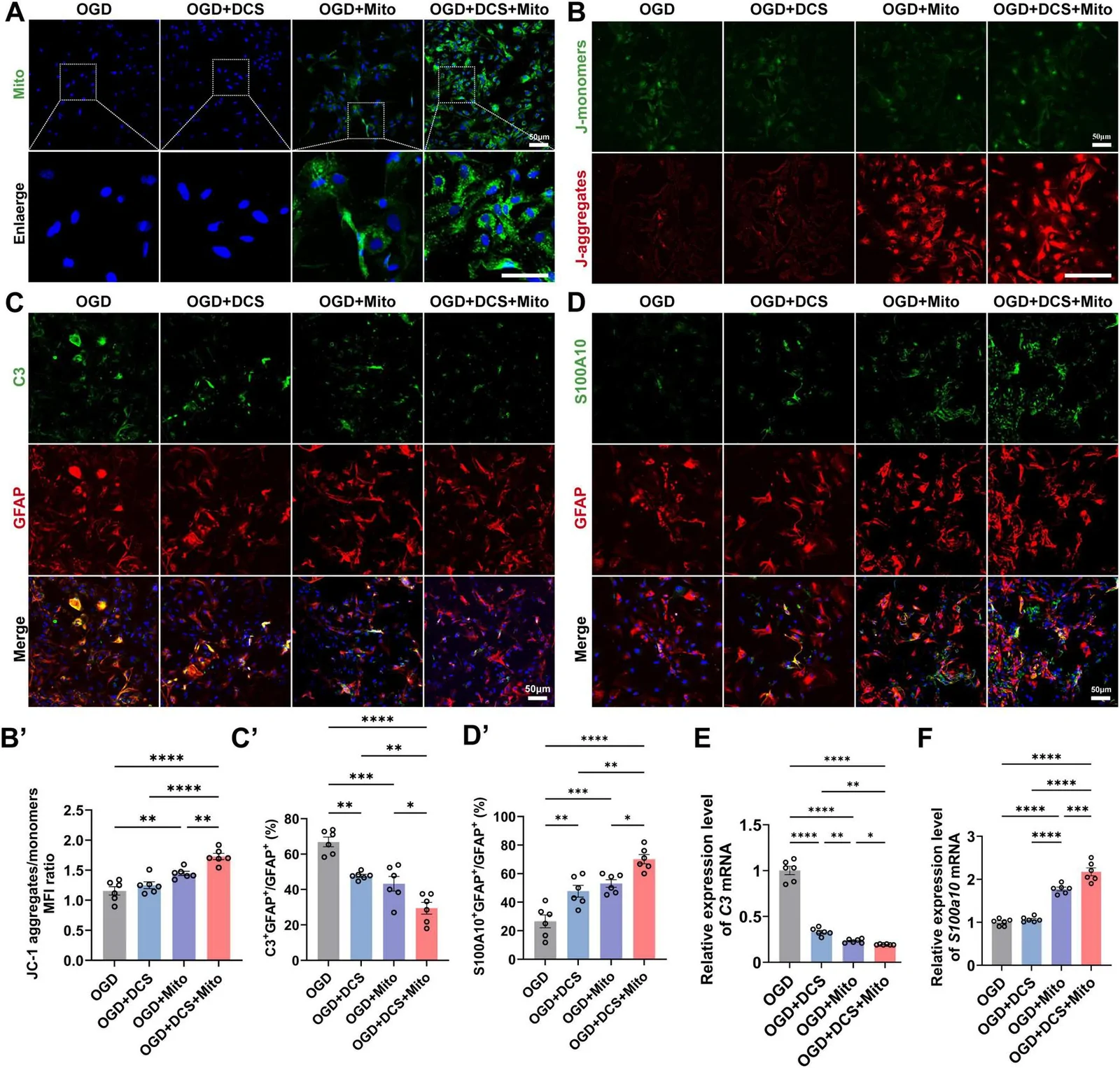
Additive effects of DCS and exogenous mitochondria in astrocytic A2 phenotype conversion *in vitro*. **(A)** Representative images of mitochondrial endocytosis in primary astrocytes under different treatments. **(B, B’)** Representative images and quantitative analysis of JC-1 fluorescent probe staining showing mitochondrial membrane potential in primary astrocytes under different treatments (*n* = 6 batches of cells, F (3, 20) = 21.19, *P* < 0.0001). **(C, C’)** Representative immunofluorescence images and quantitative analysis of C3 (marker of A1-type astrocytes) staining after OGD (*n* = 6 batches of cells, F (3, 20) = 26.27, *P* < 0.0001). **(D, D’)** Representative immunofluorescence images and quantitative analysis of S100A10 (marker of A2-type astrocytes) staining after OGD (*n* = 6 batches of cells, F (3, 20) = 23.56, *P* < 0.0001). **(E, F)** Relative mRNA expression levels of *C3* and *S100a10* in each group after OGD (*n* = 6 batches of cells, F_C3_ (3, 20) = 230.8, *P* < 0.0001, F_S100A10_ (3, 20) = 105.8, *P* < 0.0001). Scale bars = 50 μm. Data are presented as mean ± standard error of the mean (mean ± SEM). Statistical significance was indicated as follows: *P < 0.05, **P < 0.01, ***P < 0.001, ****P < 0.0001; ns, no significant difference (*P* > 0.05). OGD, oxygen-glucose deprivation. DCS, direct current stimulation. Mito, mitochondrial transplantation.

Previous studies have reported that ischemia induces two distinct phenotypes of reactive astrocytes, namely, neurotoxic A1-astrocytes, characterized by complement C3 expression, and neuroprotective A2-astrocytes, characterized by S100A10 expression. We then investigated the impact of Mito, tDCS and tDCS + Mito treatment on the A1/A2 fate of primary astrocytes treated by oxygen-glucose deprivation (OGD). As *in in vivo*, DCS significantly facilitated the Mitochondrial up-token by astrocytes after OGD (Figure 3A), In addition, highest levels of mitochondrial membrane potential were observed in astrocytes treated by OGD + tDCS + Mito, suggesting the elevated mitochondrial function in recipient cells (Figure 3B, B’). Immunostaining and quantitative analysis revealed that although DCS- and Mito-treatment alone could upregulate A2 marker and decrease A1 marker, DCS + Mito treatment led to the highest proportion of S100A10-positive A2-astrocytes and lowest portion of C3-positive A1-astrocytes among all groups (Figures 3C, C’, D, D’). In support of this, the mRNA of *C3* was most dramatically downregulated while that of *S100a10* upregulated in OGD + DCS + Mito group, as compared with OGD group, OGD + Mito group and OGD + DCS group (Figures 3E, F).

**FIGURE 4:**
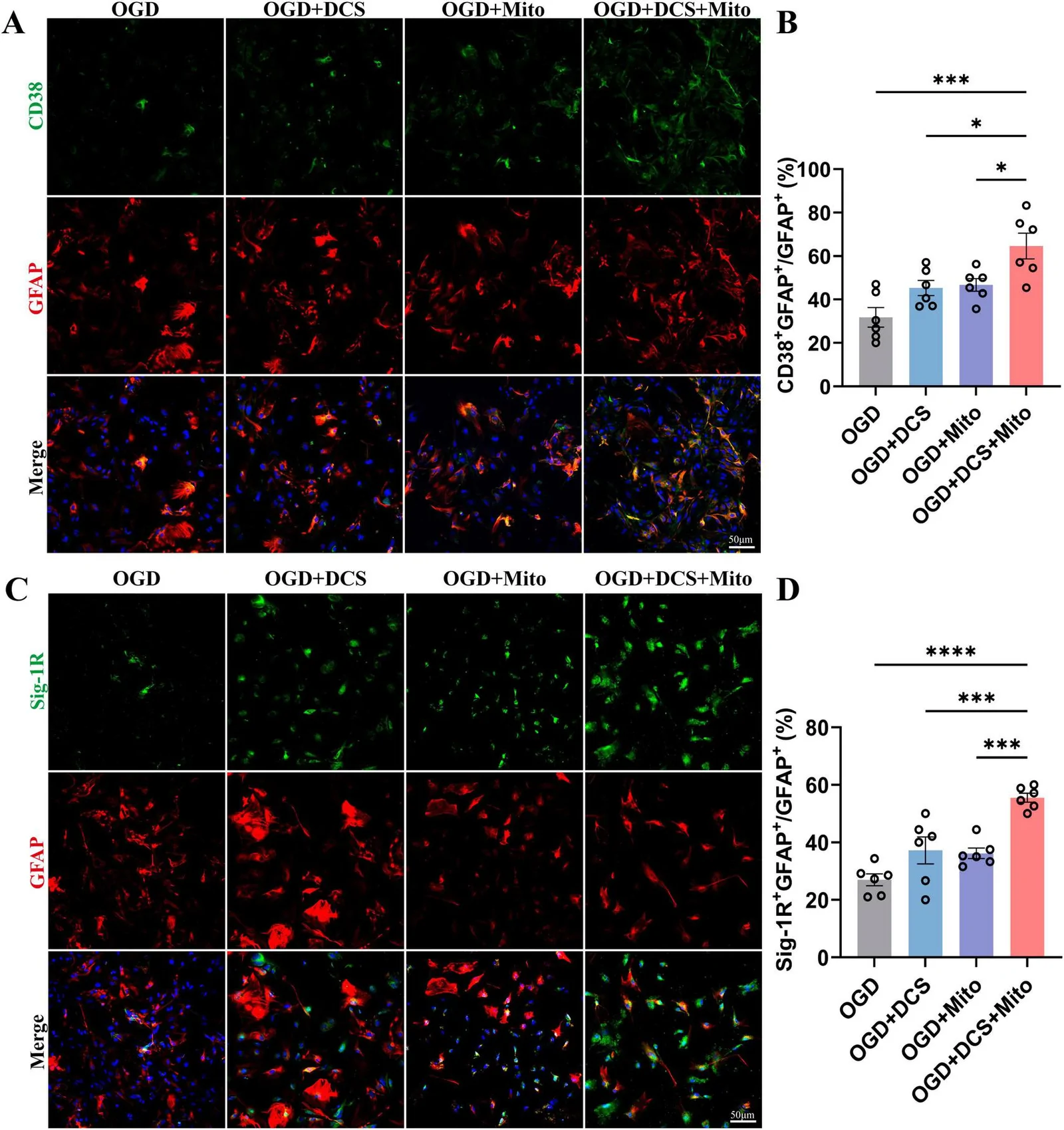
tDCS combined with mitochondrial transplantation orchestrates astrocyte A2 subtype expansion after ischemic stroke. **(A)** Representative immunofluorescence images of C3 staining at 3 dpi, and **(B)** quantitative analysis of C3^+^GFAP^+^ double-positive cells (*n* = 6 mice per group). **(C)** Relative mRNA expression levels of *C3* in cortical lesion area at 3 dpi (*n* = 3 mice per group). **(D)** Representative immunofluorescence images of S100A10 staining at 3 dpi, and **(E)** quantitative analysis of S100A10^+^GFAP^+^ double-positive cells (*n* = 6 mice per group). **(F)** Relative mRNA expression levels of *S100a10* in cortical lesion area at 3 dpi (*n* = 3 mice per group). Scale bars = 50 μm. Data are presented as mean ± standard error of the mean (mean ± SEM). Statistical significance was indicated as follows: **P < 0.01. IS, ischemia stroke. tDCS, transcranial direct current stimulation. Mito, mitochondrial transplantation.

We subsequently assessed A1-astrocytes and A2-astrocytes in the peri-infarct region at 3 days post-ischemia. The data showed that the number of A1-astrocytes significantly decreased and the number of A2-astrocytes significantly increased in the IS+tDCS+Mito treated mice compared with IS + Mito treated mice (Figures 4A, B, D, E). Furthermore, this shift was supported at the transcriptional level with *S100a10* mRNA being significantly elevated and *C3* mRNA level significantly downregulated in the IS + tDCS + Mito group (Figures 4C, F). These data demonstrated that tDCS can enhance the efficacy of mitochondrial transplantation in promoting the shift of A1-to-A2 astrocyte in the ischemic cortex.

### 3.4 Involvement of CD38 and Sig-1R in tDCS-facilitated mitochondria internalization

**FIGURE 5:**
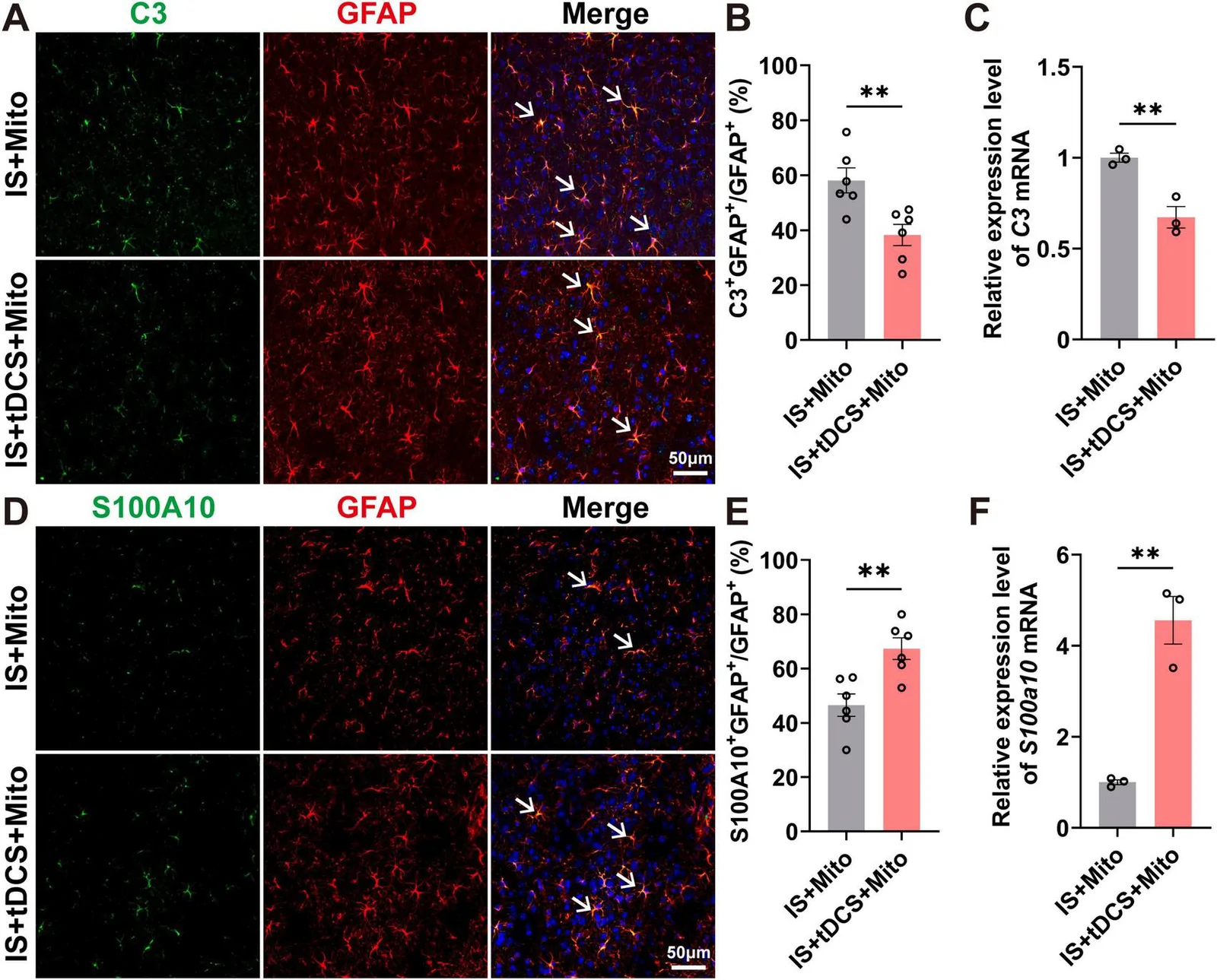
Effects of DCS and exogenous mitochondria on the expression of CD38 and Sig-1R *in vitro*. **(A)** Representative immunofluorescence images and **(B)** quantitative analysis of CD38^+^GFAP^+^ double-positive cells under different treatments (*n* = 6 batches of cells). **(C)** Representative immunofluorescence images [F (3, 20) = 9.594, *P* = 0.0004] and **(D)** quantitative analysis of Sig-1R^+^GFAP^+^ double-positive cells (*n* = 6 batches of cells, F (3, 20) = 17.90, *P* < 0.0001). Scale bars = 50 μm. Data were showed as mean ± standard error of the mean (mean ± SEM). *P < 0.05, ***P < 0.001, ****P < 0.0001. ns, no significant difference (*P* > 0.05). OGD, oxygen-glucose deprivation. DCS, direct current stimulation. Mito, mitochondrial transplantation.

We further investigated the potential molecular mechanism underlying the tDCS enhanced internalization of exogenous mitochondria after ischemia. Previous studies have demonstrated that CD38, a multi-functional protein expressed on cell surface, is a key mediator of mitochondrial transfer (Sun et al., 2019). Recently, researchers reported that astrocytic Sig-1R also played a role in facilitating mitochondrial transfer (Wang et al., 2020). Thus, we evaluated effects the expression of CD38 and Sig-1R in astrocytes under our experimental setting. In primary astrocytes subjected to OGD, immunostaining upregulation of CD38 and Sig-1R in DCS-treated and Mito-treated cells, which was further increased by combined DCS + Mito treatment (Figures 5A–D), indicating a stimulating effect of DCS on the expression of mitochondrial transfer related molecules.

**FIGURE 6:**
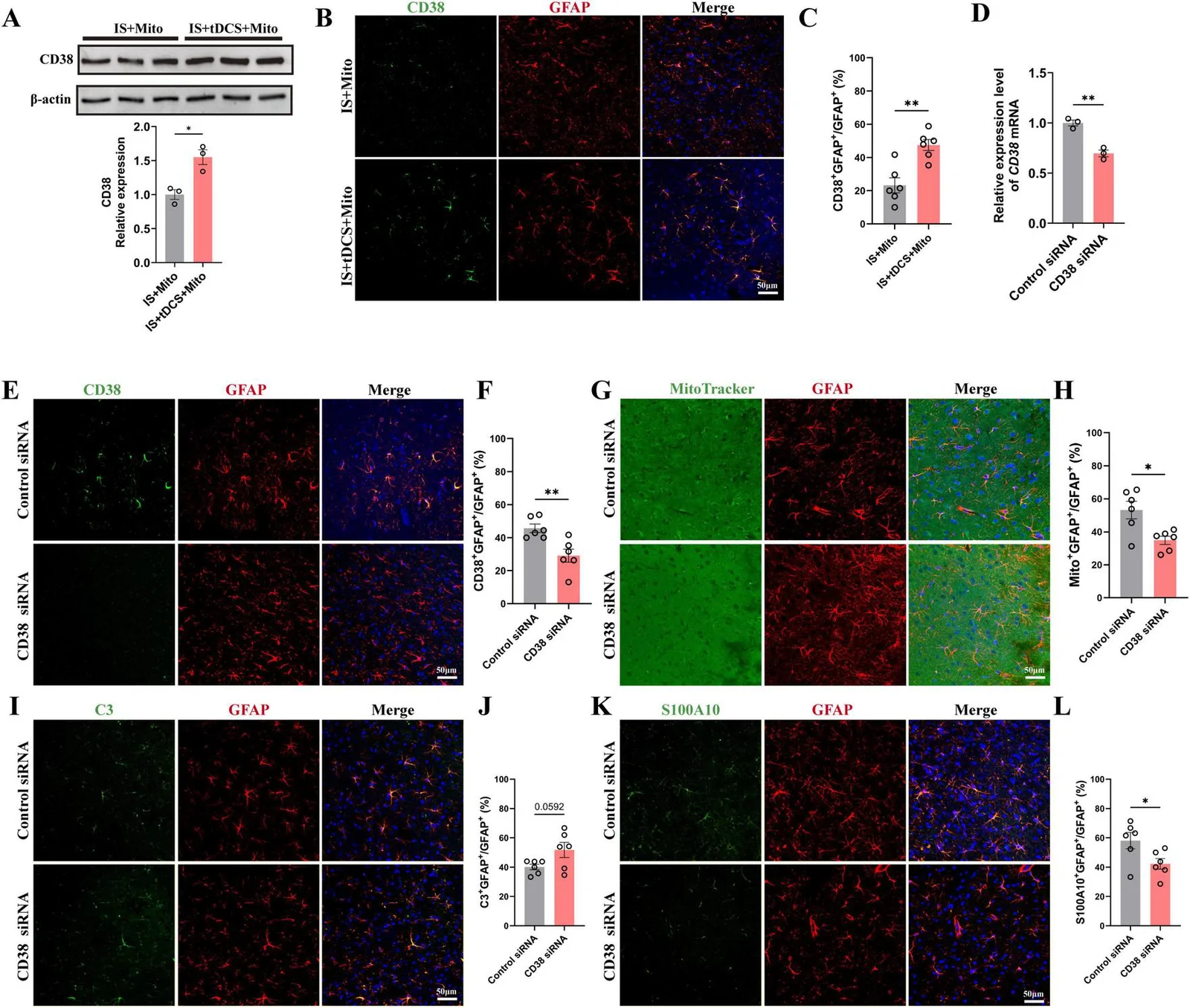
Effects of DCS and mitochondrial transplantation on the expression of CD38 and Sig-1R *in vivo*. **(A)** Western-blotting of CD38 and its quantification in ischemic cortex of IS+Mito and IS+tDCS+Mito. (*n* = 3 mice per group). **(B)** Representative immunofluorescence images and **(C)** quantitative analysis of CD38^+^GFAP^+^ double positive cells (*n* = 6 mice per group). **(D)** Relative mRNA expression levels of *CD38* in cortex at 2 days after siRNA injections (*n* = 3 mice per group). **(E)** Representative immunofluorescence images and **(F)** quantitative analysis of CD38^+^GFAP^+^ double positive cells (*n* = 6 mice per group) in control siRNA and CD38 siRNA group. **(G)** Representative immunofluorescence images and **(H)** quantitative analysis of MitoTracker^+^GFAP^+^ double positive cells (*n* = 6 mice per group) in control siRNA and CD38 siRNA group. **(I)** Representative immunofluorescence images and **(J)** quantitative analysis of C3^+^GFAP^+^ double positive cells (*n* = 6 mice per group) in control siRNA and CD38 siRNA group. **(K)** Representative immunofluorescence images and **(L)** quantitative analysis of S100A10^+^GFAP^+^ double positive cells (*n* = 6 mice per group) in control siRNA and CD38 siRNA group. Scale bars = 50 μm. Data are presented as mean ± standard error of the mean (mean ± SEM). Statistical significance was indicated as follows: *P < 0.05, **P < 0.01. IS, ischemia stroke. siRNA, small interfering RNA. tDCS, transcranial direct current stimulation. Mito, mitochondrial transplantation.

We then examined whether tDCS had a similar promoting effect on the expression of CD38 and Sig-1R *in vivo*. Western-blotting showed that the levels of CD38 (Figure 6A) and Sig-1R (Supplementary Figures 4A, B) protein in the ischemic region of IS + tDCS + Mito group were significantly higher than that in IS + Mito group. Immunostaining illustrated that more CD38/GFAP-positive and Sig-1R/GFAP-positive cells in the ischemic region of IS + tDCS + Mito group, as compared with that in IS + Mito group (Figures 6B, C, Supplementary Figures 4C, D). No significant changes of CD38 and Sig-1R expression in neurons and microglia were found between IS + Mito and IS + tDCS + Mito group (Supplementary Figure 5).

Finally, we performed loss-of-function experiments to examine whether CD38/Sig-1R signaling was involved in mitochondrial endocytosis triggered by tDCS. Before 2 days of stoke, *CD38* siRNA or control siRNA was injected into ishchemic cortex. Immunostaining and qPCR results showed that CD38 expression in the peri-infarct cortex was successfully downregulated (Figures 6D–F). Mice with *CD38* siRNA treated showed a significant reduction in astrocyte mitochondria endocytosis, as well as a decrease of A2 astrocytes (Figures 6G–L).

In together, these data indicated that tDCS might facilitate mitochondrial internalization by the up-regulation of CD38 and Sig-1R.

## 4 Discussion

Our study investigated the therapeutic impact of combined tDCS and mitochondrial transplantation on protecting the brain against ischemic injury. Although tDCS or mitochondrial transplantation alone can significantly reduce infarct volume and improve neurological outcomes following ischemic stroke, the combination of tDCS and mitochondrial transplantation was superior in improving functional recovery. Mechanistically, we found that tDCS promote the efficient internalization of exogenous mitochondria by astrocytes. This enhanced uptake inhibited neurotoxic A1 astrocytes, and promoted neuroprotective A2 astrocytes on the other side. Furthermore, this process may be mediated by the upregulation of key signaling molecules CD38 and Sig-1R in astrocytes.

Consistent with previous studies, our results showed that either tDCS or mitochondrial transplantation can significantly reduce infarct volume and accelerate motor functional recovery following cerebral ischemia. This substantiates the feasibility of combined therapy with tDCS and mitochondria transplantation. Notably, we observed that combined tDCS with mitochondria transplantation decreased infarction volumes and improved functional outcomes significantly to a greater extent than other groups, suggesting that the combined treatment may allow the maximization of functional recovery in the post-stroke mice models. Previous research has established that transplanted exogenous mitochondria could be internalized by neural cells, including neurons and glial cells (Zhao et al., 2021). Our data showed a similar pattern of mitochondrial internalization. What is interesting is that tDCS only stimulated the capacity of astrocytes, not neurons, in up-taking exogenous mitochondria. This does not imply that neuronal mitochondrial incorporation is ineffective. Rather, to certain extent, it is in line with previous view that astrocyte membrane is sensitive to tDCS stimulation (Ruohonen and Karhu, 2012; Gellner et al., 2016; Monai and Hirase, 2018). Monai et al. showed that tDCS induces large-amplitude Ca^2 +^ surges specifically in astrocytes, with no obvious changes in neuronal local field potentials (Monai et al., 2016). The finding that tDCS does not enhance astrocytic uptake of exogenous mitochondria in non-ischemic normal mice indicates that its regulatory effect on mitochondrial internalization depends on the ischemic microenvironment, such as the up-regulation of CD38 under ischemia (Hayakawa et al., 2016).

Under the inflammatory conditions induces by cerebral ischemia, astrocytes are activated to detrimental A1 phenotype and beneficial A2 phenotype (Escartin et al., 2021; Lu and Wen, 2025). Our findings demonstrate that individual application of either tDCS or mitochondrial transplantation can downregulate the expression of the A1 astrocyte marker C3 and upregulating the expression of the A2 marker S100A10, which was consistent with previous report that tDCS can exerts neuroprotective effect by shifting reactive astrocytes from A1 phenotype to A2 phenotype via NF-κB/NLRP3/IL-18 (Baba et al., 2009; Xu et al., 2018; Geng et al., 2023; Liu et al., 2025). Exogenous mitochondria transplantation can up-regulate astrocytic BDNF expression in traumatic brain injury (Zhao et al., 2021). Our data further indicate that mitochondria transplantation may has a regulatory effect on the phenotypic transform of astrocytes. More importantly, the combination of tDCS with mitochondria transplantation increased the percentage of beneficial phenotype astrocytes, indicating a phenotype changing of this therapeutic strategy.

Emerging evidences from *in vitro*, animal and clinical studies suggests that mitochondrial transplantation may be a promising therapeutic strategy for various central nervous system disorders, including spinal cord injury, schizophrenia, Parkinson’s disease, ischemic stroke (Nakamura et al., 2020). The cellular uptake of exogeneous mitochondria is essential for the outcome of these treatments. Endocytosis, nano-tunnel formation and gap junctions are proposed as the potential mechanism of intercellular mitochondrial transfer (Geng et al., 2023). Considering the size of mitochondria, endocytosis maybe the most efficient way for a cell to uptake exogenous mitochondria. Sun et al. proposed an actin-dependent endocytosis pathway, namely NAD^+^-CD38-cADPR-Ca^2+^ signaling pathway, to mediate the neuron-to-astrocyte mitochondrial transfer (Sun et al., 2019). CD38 catalyzes the cyclization of extracellular NAD^+^ to generate intracellular cyclic ADP-ribose (cADPR), then triggering the release of Ca^2+^. The released Ca^2+^ concentrated near the cell membrane that promote endocytosis by initiating F-actin polymerization and facilitating cytoskeletal remodeling, which results in the internalization of exogenous mitochondria (Zhang et al., 2019). Our results found that both *in vitro* and *in vivo*, the tDCS/DCS + Mito group exhibited an increased CD38 expression in astrocytes, and suppression of CD38 expression by short interfering RNA reduced astrocyte mitochondrial endocytosis induced by tDCS, indicate that tDCS may upregulate CD38 expression to promote mitochondrial internalization. In addition, Sig-1R, an endoplasmic reticulum chaperone protein which facilitate CD38 expression, was also up-regulated by tDCS/DCS + Mito treatment. Given that Sig-1R can associate with IP3R and Rac1 to form a multiprotein complex for maintaining mitochondrial integrity and regulating mitochondrial-actin interaction (Shi et al., 2021), it is possible that CD38 and Sig-1R may mediate the increased mitochondrial endocytosis effects of tDCS. The detailed mechanism how tDCS stimulated the expression of CD38 and Sig-1R is worthy to be explored in the future.

Several limitations should be acknowledged of this study. First, only male mice were used, precluding assessment of sex-dependent differences. Second, histological analyses were performed only at 3 days post-injury, leaving the durability of cellular effects unclear. Third, the photothrombotic model does not fully recapitulate human stroke pathophysiology, particularly reperfusion dynamics and collateral circulation. Fourth, mitochondria were isolated from liver rather than brain tissue. Albeit that mitochondria from different tissues may differ metabolically, recent comprehensive reviews have highlighted liver tissue as one of the primary sources for mitochondria isolation in transplantation studies targeting the brain (Girgenti et al., 2025; Kubat et al., 2025). Future studies incorporating both sexes, extended observation periods, multiple stroke models, and comparative analyses of differernt mitochondrial sources are warranted to generalize our findings. Despite these limitations, our study provides proof-of-concept evidence for additive neuroprotection combining tDCS with mitochondrial transplantation.

## 5 Conclusions

In summary, the present study demonstrated a additive effect that tDCS can reinforce the therapeutic efficacy of mitochondrial transplantation in a mouse model of ischemic stroke. Mechanistically, tDCS promotes the internalization of exogenous mitochondria in astrocytes via upregulating CD38/Sig-1R and promoted the A2 phenotype of reactive astrocytes. Our findings provide preclinical evidence that combination of tDCS and mitochondrial transplantation may be a promising translational strategy for stroke intervention and rehabilitation.

## Data Availability

The original contributions presented in the study are included in the article/Supplementary material, further inquiries can be directed to the corresponding author.
