# Supplementary material for: Additive protection of transcranial direct current stimulation and mitochondrial transplantation on ischemic stroke via inducing A2 astrocytes
Source: Front Mol Neurosci. 2026 Sep 9;19:1855078. doi: 10.3389/fnmol.2026.1855078 (PMC13599403; doi:10.3389/fnmol.2026.1855078)

Western blotting

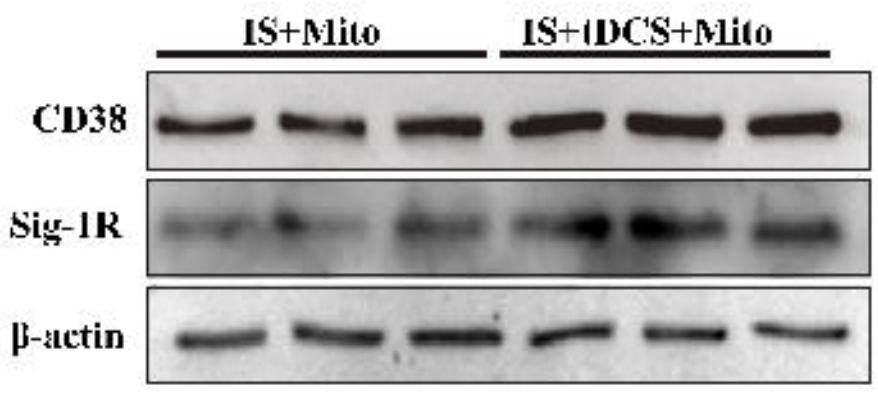

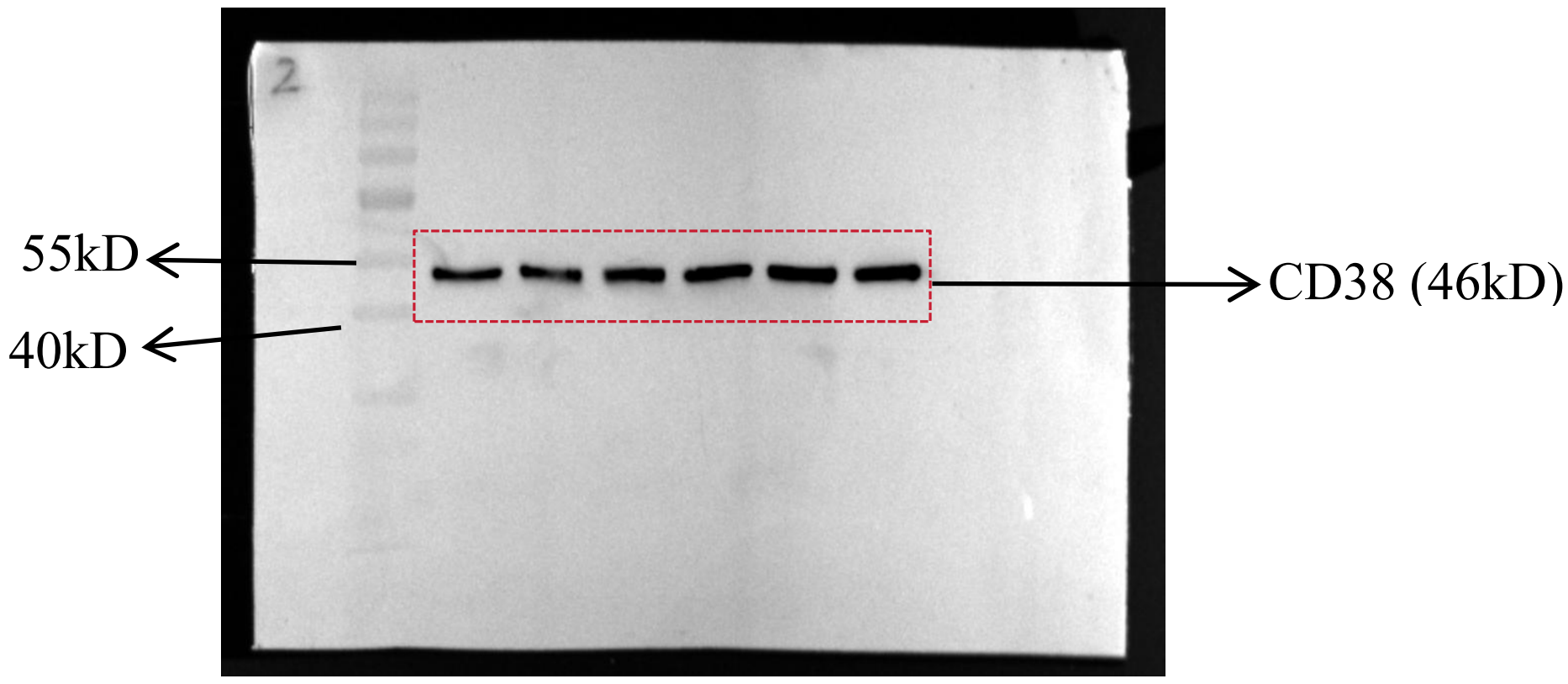

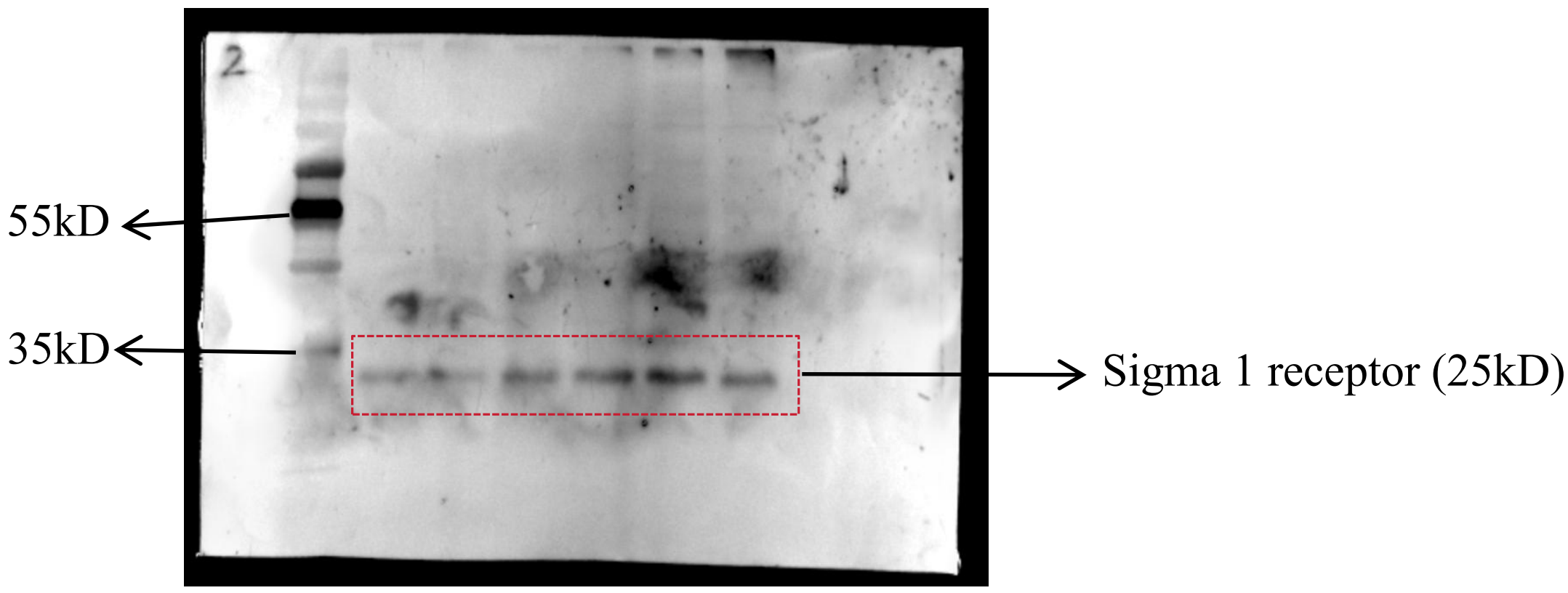

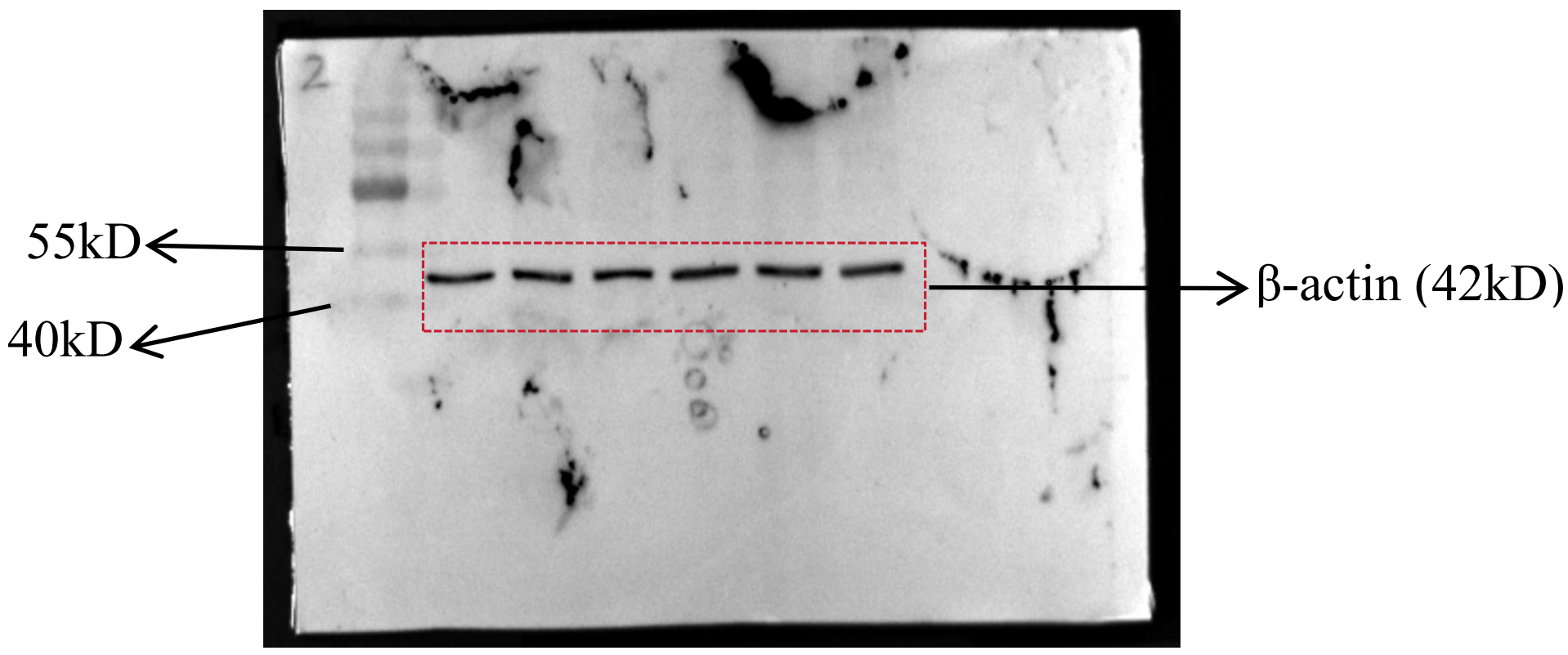

Supplement: Supplementary file 1 [file Data_Sheet_1.pdf]
