# Supplementary material for: Additive protection of transcranial direct current stimulation and mitochondrial transplantation on ischemic stroke via inducing A2 astrocytes
Source: Front Mol Neurosci. 2026 Sep 9;19:1855078. doi: 10.3389/fnmol.2026.1855078 (PMC13599403; doi:10.3389/fnmol.2026.1855078)
Supplement: Supplementary file 2 [file Supplementary_file_1.docx]

Supplementary Material

Additive protection of transcranial direct current stimulation and mitochondrial transplantation on ischemic stroke via inducing A2 astrocytes

**Yaomin Guo^1#^, Enming Kang^2,3#^, Min Li^2,3,4#^, Taozhi Wang^5^, Zhongqing Sun^1^, Yazhou Wang^2,3*^, Wen Jiang^1*^, Jiang Li^6*^**

^1^Department of Neurology, Xijing Hospital, Fourth Military Medical University, Xi'an, Shaanxi, 710032, China

^2^Department of Neurobiology, School of Basic Medicine, Fourth Military Medical University, Xi'an, Shaanxi, 710032, China

^3^The Shaanxi Province Key Laboratory of Brain Function Analysis and Modulation, Xi'an, Shaanxi, 710032, China

^4^Department of Neurobiology, Medical College of Yan' an University, Yan' an 716000, China

^5^Department of Anesthesiology and Perioperative Medicine, Xijing Hospital, The Fourth Military Medical University, Xi'an, Shaanxi, 710032, China

^6^Department of Neurosurgery, Tangdu Hospital, Fourth Military Medical University, Xi’an, Shaanxi, 710038, China

**Supplementary Figures**

**Supplementary Figure 1.** Preparation of mitochondria for transplantation. (A) Bright-field microscopy of MitoTracker-labeled freshly isolated mitochondria. (B) Activity of respiratory chain complexes I-Ⅳ of freshly isolated mitochondria (n = 3 per group). Scale bars = 50μm.

**Supplementary Figure 2.** Histological images of TUNEL staining showing robust apoptosis in the margin area of ischemic lesion at 3 dpi. IS, ischemia.

**Supplementary Figure 3.** Representative histological images of Nissel staining of the ischemic region in different groups. IS, ischemia. tDCS, transcranial direct current stimulation. Mito, mitochondrial transplantation.

**Supplementary Figure 4.** (A) Western-blotting of Sig-1R and its quantification in ischemic cortex of IS+Mito and IS+tDCS+Mito. (n = 3 mice per group). The β-actin loading control shown is from the same membrane as used for Protein CD38 in Figure 6A. (B) Representative immunofluorescence images and (C) quantitative analysis of Sig-1R^+^GFAP^+^ double positive cells (n = 6 mice per group). Scale bars = 50μm. Data are presented as mean ± standard error of the mean (mean ± SEM). Statistical significance was indicated as follows: **P* < 0.05, *****P* < 0.0001. IS, ischemia stroke. tDCS, transcranial direct current stimulation. Mito, mitochondrial transplantation. Sig-1R, Sigma 1 receptor.

**Supplementary Figure 5.** Expression of CD38 and Sig-1R in ischemic cortex treated with mitochondria, or combination of tDCS and mitochondria. (A, A’) Representative immunofluorescence images and quantitative analysis of CD38^+^NeuN^+^ double positive cells. (B, B’) Representative immunofluorescence images and quantitative analysis of Sig-1R^+^NeuN^+^ double positive cells. (C, C’) Representative immunofluorescence images and quantitative analysis of CD38^+^Iba-1^+^ double positive cells. (D, D’) Representative immunofluorescence images and quantitative analysis of Sig-1R^+^Iba-1^+^ double positive cells. (n = 3 mice per group). Scale bars = 50μm. Data are presented as mean ± standard error of the mean (mean ± SEM). ns, no significant difference (*P* > 0.05). IS, ischemia. tDCS, transcranial direct current stimulation. Mito, mitochondrial transplantation.
